# RETRACTION: Comparative Effectiveness of Pericapsular Nerve Group Block Versus Fascia Iliac Compartment Block on Postoperative Wound Pain Management in Patients Undergoing Hip Fracture Surgery: A Systematic Review and Meta‐Analysis

**DOI:** 10.1111/iwj.70523

**Published:** 2025-04-18

## Abstract

**RETRACTION:** MouZ. , XiangL. , and NiY. , “Comparative Effectiveness of Pericapsular Nerve Group Block Versus Fascia Iliac Compartment Block on Postoperative Wound Pain Management in Patients Undergoing Hip Fracture Surgery: A Systematic Review and Meta‐Analysis,” International Wound Journal 21, no. 2 (2024): e14637, 10.1111/iwj.14637.38332471 PMC10853491

The above article, published online on 08 February 2024, in Wiley Online Library (http://onlinelibrary.wiley.com/), has been retracted by agreement between the journal Editor in Chief, Professor Keith Harding; and John Wiley & Sons Ltd. Following an investigation by the publisher, all parties have concluded that this article was accepted solely on the basis of a compromised peer review process. The editors have therefore decided to retract the article. The authors did not respond to our notice regarding the retraction.

**RETRACTION:** Z. Mou , L. Xiang , and Y. Ni , “Comparative Effectiveness of Pericapsular Nerve Group Block Versus Fascia Iliac Compartment Block on Postoperative Wound Pain Management in Patients Undergoing Hip Fracture Surgery: A Systematic Review and Meta‐Analysis,” International Wound Journal 21, no. 2 (2024): e14637, 10.1111/iwj.14637.38332471 PMC10853491

The above article, published online on 08 February 2024, in Wiley Online Library (http://onlinelibrary.wiley.com/), has been retracted by agreement between the journal Editor in Chief, Professor Keith Harding; and John Wiley & Sons Ltd. Following an investigation by the publisher, all parties have concluded that this article was accepted solely on the basis of a compromised peer review process. The editors have therefore decided to retract the article. The authors did not respond to our notice regarding the retraction.

